# When Something Seems Amiss: Radiology-Pathology Correlation of Metaplastic Breast Cancer

**DOI:** 10.7759/cureus.8239

**Published:** 2020-05-22

**Authors:** Sonia Andreou, Erik Soule, Deidra Long, Bharti Jasra, Smita Sharma

**Affiliations:** 1 Surgery, University of Florida College of Medicine, Jacksonville, USA; 2 Interventional Radiology, University of Florida College of Medicine, Jacksonville, USA; 3 Pathology, University of Florida College of Medicine, Jacksonville, USA; 4 Radiology, University of Florida College of Medicine, Jacksonville, USA

**Keywords:** spindle cell metaplastic breast cancer, metaplastic breast cancer, breast mri, immunotherapy, sampling bias, intratumoral heterogeneity, radiomics, p63

## Abstract

Metaplastic breast cancer is difficult to diagnose, resistant to conventional treatment, and biologically aggressive. A suspicious timeline and discordance between imaging findings and histopathologic tissue diagnosis should trigger additional workup. New, large lesions or rapidly growing lesions with complex echogenicity on ultrasound warrant correlation with image-guided biopsy for a definitive diagnosis. Lesions that appear aggressive on imaging, with negative biopsy findings, may represent false negatives due to sampling bias from intratumoral heterogeneity. In such cases, it may be advisable to obtain an excisional biopsy. These tumors are known to progress even with neoadjuvant chemotherapy. Immunotherapy, however, may be effective even for metastatic disease. A multidisciplinary approach and a high index of suspicion may, therefore, confer survival benefits in circumstances where the imaging phenotype does not fit with the timeline or pathologic diagnosis. This report describes five cases of metaplastic breast cancer diagnosed at our institution to highlight the importance of a timely and accurate diagnosis of this rare but aggressive breast malignancy.

## Introduction

Metaplastic breast cancer (MBC) comprises a heterogeneous group of rare, biologically aggressive malignancies. Pathologically, they are ductal carcinomas that undergo a metaplastic transformation into a nonglandular growth pattern. In contrast to the more common breast cancer subtypes, which tend to metastasize to the axillary nodes, MBC tends to metastasize to distant sites such as the brain and lungs [1]. These cancers typically do not express estrogen receptor, progesterone receptor, or human epidermal growth factor receptor 2 (HER2) but may overexpress programmed death-ligand 1 (PD-L1). This may result in a dramatic response to newly developed immunotherapy medications, even for metastatic disease that has traditionally portended a dismal prognosis [2]. Therefore, it is critical to identify these patients early on in the course of the disease so that they may receive individualized treatment and the best possible chance for survival.

Early diagnosis presents a challenge, as the radiologic phenotypes of MBC are diverse and may mimic benign lesions or other invasive breast carcinomas. There is no mammographic or sonographic appearance that reliably predicts the presence of MBC [3-4]. High signal intensity on T2-weighted magnetic resonance imaging (MRI) related to the necrotic component of the tumor, however, may be a useful imaging marker [5]. Additional challenges in the diagnosis of MBC include histologic heterogeneity among the subtypes of MBC. An example of this is spindle cell tumors, which may be mistaken for fibromatosis [6]. The biopsy may also be confounded due to sampling bias from intratumoral heterogeneity [7].

## Case presentation

Case 1

A 47-Year-Old Female With a Palpable Right Breast Mass

**Figure 1:**
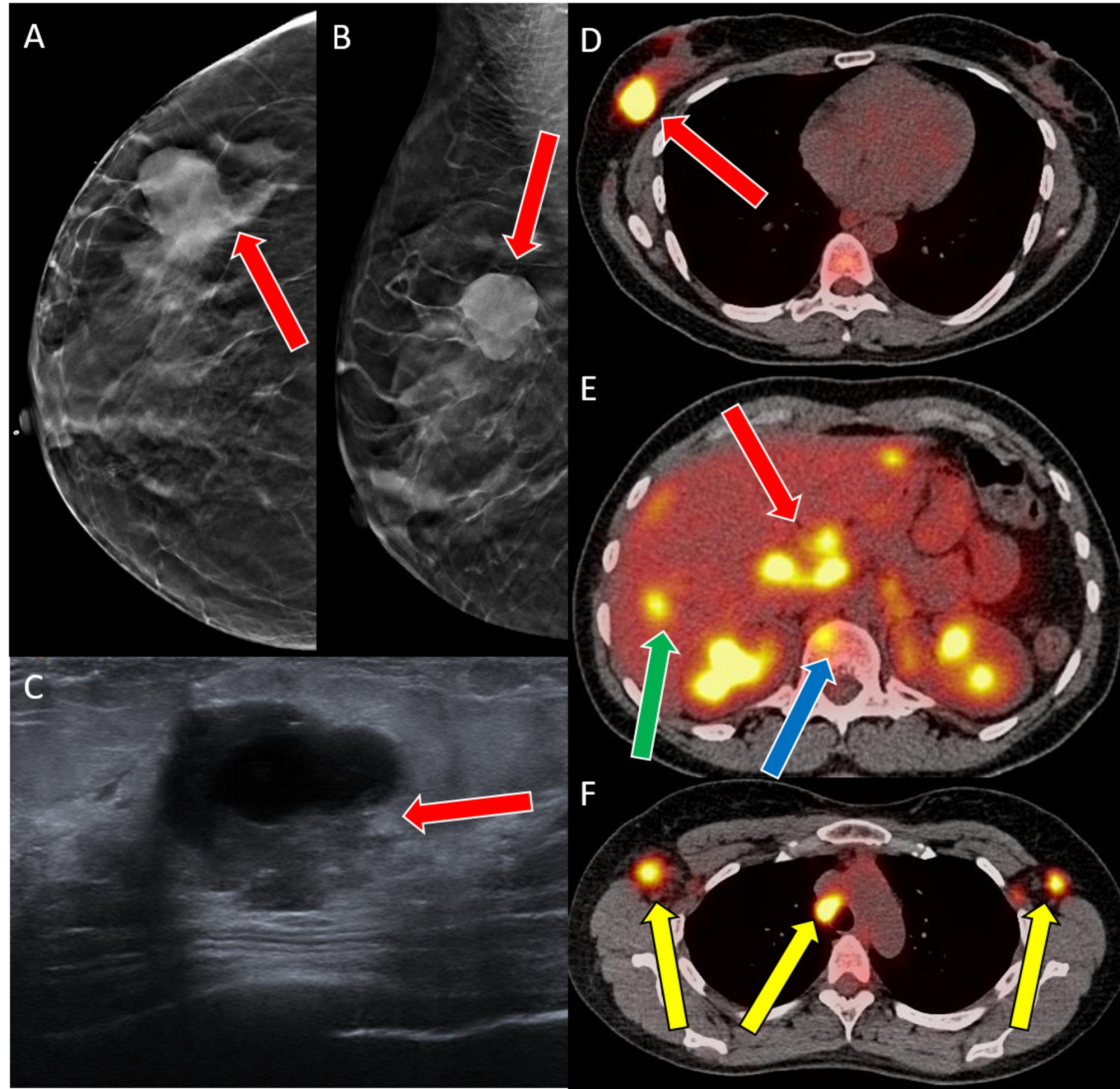
Case 1 imaging findings A, B: Craniocaudal and mediolateral oblique tomosynthesis views of the right breast show an oval high-density mass (red arrows) with partially spiculated margins. C: Transverse ultrasound shows an oval mixed solid and cystic mass (red arrow) with partially indistinct margins. D-F: PET demonstrates (D) a hypermetabolic right breast mass (red arrow), (E) porta-hepatis lymphadenopathy (red arrow), hepatic (green arrow) and bony metastasis (blue arrow), (F) mediastinal and bilateral axillary lymphadenopathy (yellow arrows). PET: positron emission tomography

Diagnostic mammogram and ultrasound showed a 4.7 cm right breast mass (Figures 1A-1C). A subsequent positron emission tomography (PET) scan showed metastatic disease to the bilateral axilla, porta hepatis, vertebrae, and liver (Figures 1D-1F).

**Figure 2:**
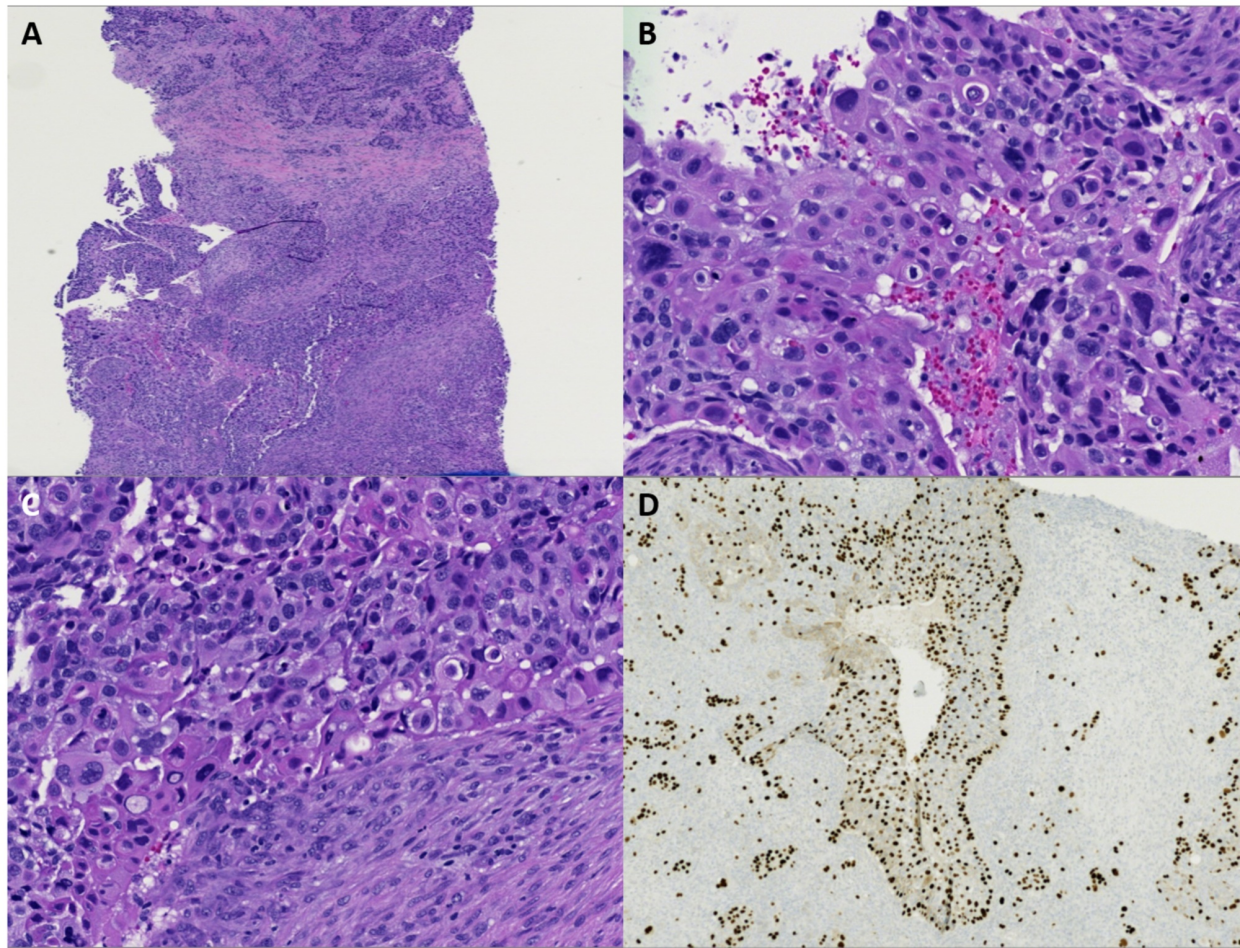
Case 1 histopathology findings A: Low power view of metaplastic carcinoma, squamous cell carcinoma subtype. B: Poorly differentiated, large polygonal cells with abundant, eosinophilic cytoplasm (10x magnification). C: Keratinized cells with numerous atypical mitoses and pyknotic cells present (10x magnification). D: P63 is a nuclear immunostain, which highlights the atypical squamous cells, further supporting the diagnosis of MBC, squamous cell type. MBC: metaplastic breast cancer

The highly aggressive presentation with metastatic disease and lymphadenopathy, as well as the histology of the tumor, is consistent with a high-grade carcinoma. Ultrasound-guided core biopsy of the right breast mass and right axilla demonstrated metaplastic breast carcinoma with squamous differentiation (Figures 2A-2B). Clear squamous differentiation is evidenced by keratinization and intercellular bridges (Figure 2C). Additionally, a positive immunohistochemical stain for P63 is consistent with metaplastic carcinoma with squamous differentiation (Figure 2D). This patient opted to eschew aggressive care and elected for hospice treatment. Although this type of aggressive malignancy has traditionally portended a dismal prognosis, in the near future, immunotherapy may be a potentially curative treatment option for advanced MBC.

Case 2

A 44-Year-Old Female With a Rapidly Enlarging Left Breast Mass

**Figure 3:**
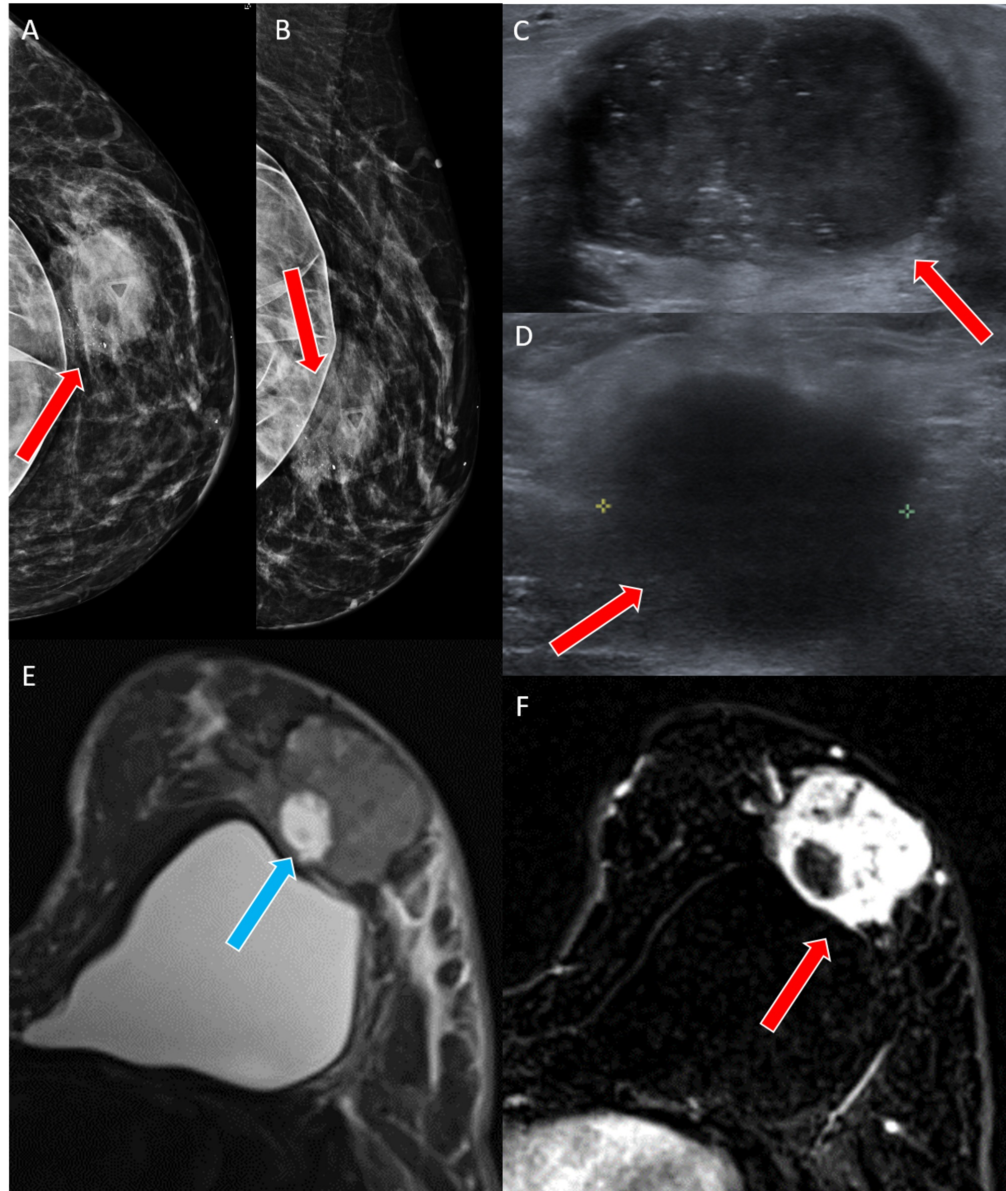
Case 2 imaging findings A, B: Craniocaudal and mediolateral oblique tomosynthesis views of the left breast show a round mass with partially obscured margins and pleomorphic calcifications (red arrows) in the central left breast corresponding to the area of palpable concern. The marker clip is from a reportedly prior benign biopsy. C: Ultrasound shows an oval, circumscribed hypoechoic mass (red arrow) with echogenic calcifications. D: A pathologic hypoechoic lymph node (red arrow) is seen in the left axilla on ultrasound. E, F: MRI shows a heterogeneously enhancing mass (red arrows) with an area of T2 hyperintensity (blue arrow) consistent with necrosis.

A 44-year-old female presented with an erythematous and painful left breast mass measuring 6.0 cm x 3.1 cm on mammography and ultrasound (Figures 3A-3D). A breast MRI revealed diffuse abnormal T2 hyperintense edema and skin thickening (Figures 3E-3F).

**Figure 4:**
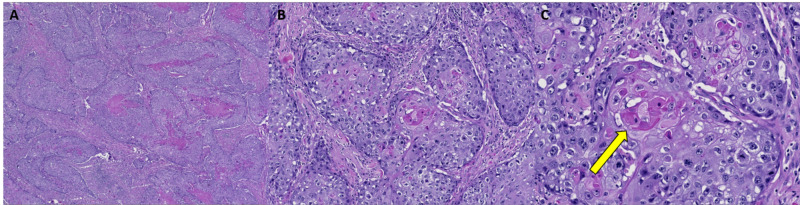
Case 2 histopathology findings A: Low magnification image of metaplastic carcinoma with squamous differentiation and extensive necrosis. B: Area of moderately differentiated squamous cell carcinoma (10x). C: High magnification of image B, highlighting distinct keratinization of cells (yellow arrow) with surrounding cells that have a high nuclear to cytoplasmic ratio, abundant cytoplasm, and frequent mitoses (20x).

She reported two prior breast biopsies, 10 and six years prior to presentation, which were benign. Ultrasound-guided core biopsies of the mass and enlarged lymph node were performed and initially reported as invasive ductal carcinoma with squamoid differentiation. The patient underwent neoadjuvant chemotherapy. However, when the cancer progressed and showed further inflammatory changes, the mass was re-biopsied. At that time, the correct diagnosis of metaplastic breast carcinoma with squamous differentiation was established. Histology revealed very high-grade pleomorphic tumor cells with extensive necrosis and areas of keratinization (Figures 4A-4C).

Case 3

A 49-Year-Old Female for a Six-Month Follow-Up of a Benign Left Breast Biopsy

**Figure 5:**
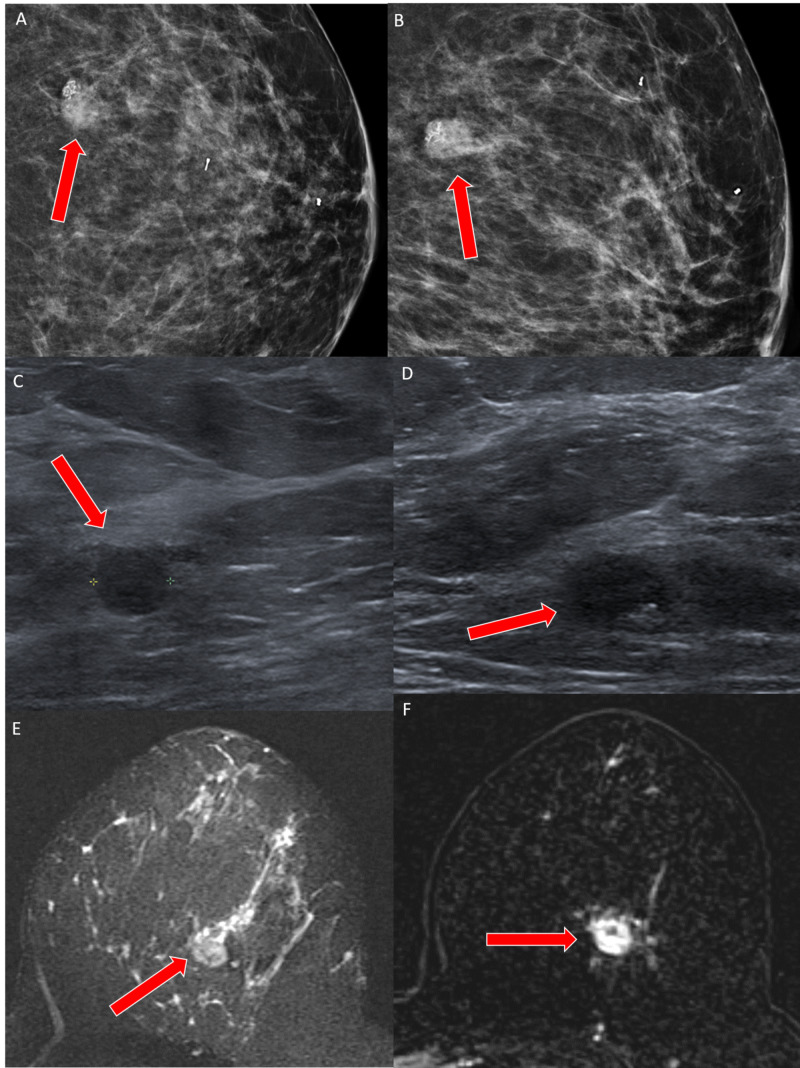
Case 3 imaging findings A, B: Craniocaudal and mediolateral oblique tomosynthesis views of the left breast show a new, round, equal density mass (red arrows) with partially obscured margins and coarse calcifications. C, D: Ultrasound demonstrates a circumscribed round hypoechoic mass (red arrows) with echogenic calcifications and partially obscured and microlobulated margins. E, F: MRI shows a round, T2, hyperintense, homogeneously enhancing mass (red arrows). MRI: magnetic resonance imaging

A 49-year-old female presented with an area of palpable concern on her left breast with tenderness. A previous biopsy clip was noted on the screening mammogram in the left breast. Ultrasound-guided core biopsy was performed, which was reported as benign, revealing only stromal sclerosis, skeletal muscle fibers, and mild periductal chronic inflammatory cells. Six months later, a repeat diagnostic mammogram revealed a new, ill-defined mass with very dense coarse calcifications (Figures 5A-5B). Breast ultrasound was then performed, which showed a small circumscribed hypoechoic mass with partially obscured and microlobulated margins (Figures 5C-5D). Ultrasound-guided core biopsy was performed, yielding the diagnosis of MBC. Breast MRI was also performed at this time, revealing a round T2 hyperintense, homogenously enhancing mass (Figures 5E-5F).

**Figure 6:**
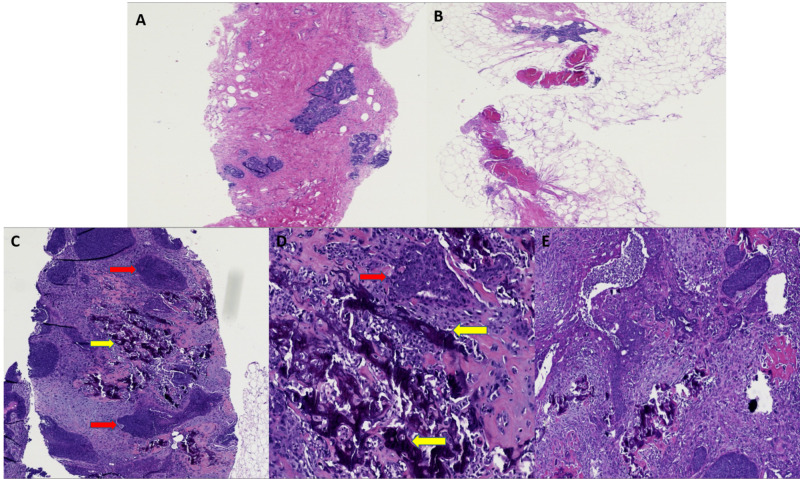
Case 3 histopathology findings A: Initial ultrasound-guided core biopsy, which revealed benign mammary tissue with stromal sclerosis and mild periductal inflammation, along with fragments of benign skeletal muscle. B: No areas suspicious for malignancy were present in the biopsy material submitted. C-D: Second ultrasound-guided core biopsy showing high-grade invasive ductal carcinoma (red arrows) with intermingled areas of osseous differentiation (yellow arrows). E: Representative section from a mastectomy with similar findings of metaplastic carcinoma with osseous metaplasia.

Histologically, the first biopsy demonstrated benign findings (Figures 6A-6B). The second biopsy revealed a tumor highlighted by clear nests of high-grade carcinoma (Figures 6A-6D). Admixed with the carcinoma, there was also a very discrete sarcomatous component to the tumor, which contained malignant bone formation. The coarse calcifications observed radiologically corresponded to malignant osteoid. This presentation of a carcinoma accompanied by sarcomatous elements is diagnostic of metaplastic carcinoma with heterologous differentiation. The tumor profile was triple-negative, and the patient elected for a bilateral mastectomy with adjuvant chemotherapy. The mastectomy specimen revealed infiltrating mammary carcinoma, poorly differentiated, metaplastic type with osseous metaplasia. The malignancy was high-grade (Nottingham grade 3) with pronounced nuclear pleomorphism and moderate mitotic activity. The margins were uninvolved and angiolymphatic invasion was not identified. The previous biopsy sites demonstrated sclerosed papilloma and usual ductal hyperplasia. The final pathologic stage was pT1cN0.

Case 4

A 20-Year-Old Female With a Right Breast Palpable Mass

**Figure 7:**
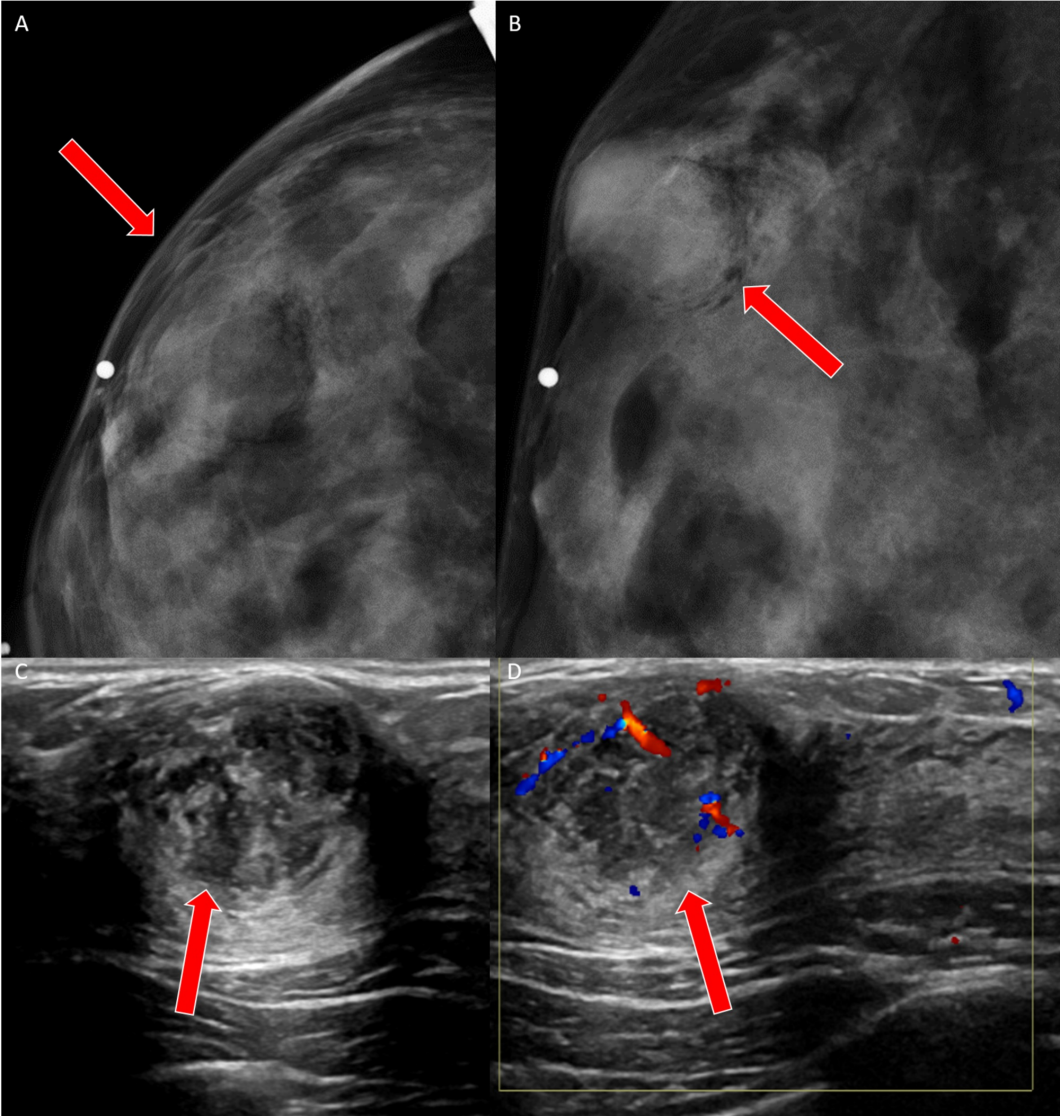
Case 4 imaging findings A, B: Right diagnostic mammogram shows a round circumscribed mass (red arrows) in the upper outer quadrant corresponding to the area of palpable concern. C, D: The ultrasound shows a round, circumscribed mass (red arrows) with heterogeneous echotexture and internal vascularity.

A 20-year-old female with no significant past medical history presented with a palpable mass in her right breast. The mammogram revealed an oval equal density mass with obscured margins measuring up to 2.1 cm but the sensitivity of the mammography was limited due to extremely dense breasts (Figures 7A-7B). Ultrasound revealed an oval, heterogeneously hypoechoic mass with indistinct margins and posterior acoustic enhancement (Figures 7C-7D). The initial ultrasound-guided core biopsy was reported as a fibroepithelial neoplasm with abundant stroma. This was thought to represent a benign phyllodes tumor but complete excision was recommended.

**Figure 8:**
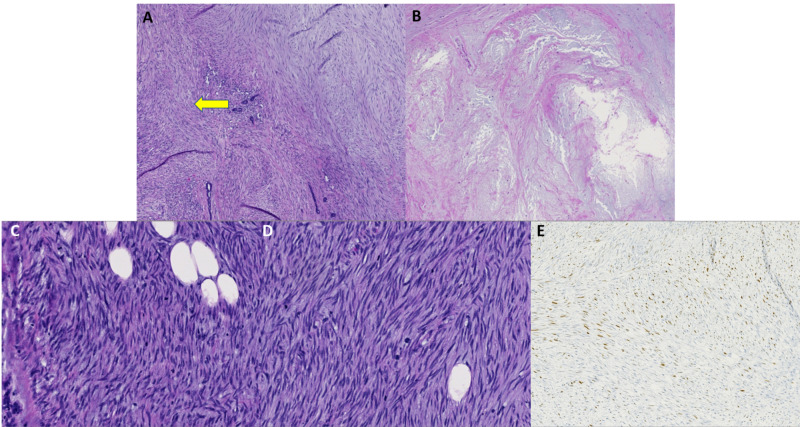
Case 4 histopathology findings A: Low power magnification of a spindle cell lesion with entrapment of normal mammary glandular structures (yellow arrow), 5x. B: Low magnification demonstrating necrosis and degeneration of the tumor, 5x. C-D: High power images emphasizing the sweeping fascicles of spindle cells, in the classic herringbone pattern, along with frequent mitotic figures and abnormal nuclei, 20x. E: P63 immunostain showing strong, nuclear positivity in the spindle cells, 10x

Three months later, the breast lump continued to grow rapidly, and excision was performed. Evaluation of that specimen revealed the correct diagnosis of metaplastic breast carcinoma, spindle cell variant. Histologically, the tumor consisted of bland spindle cells with mild-moderate pleomorphism and several mitotic figures, concerning for either a low-grade sarcoma versus a metaplastic carcinoma. Extensive central necrosis was observed. Staining for P63, as well as focal staining for cytokeratins along with the histology of the tumor, was consistent with a low-grade fibromatosis-like metaplastic carcinoma (Figures 8A-8D).

Case 5

A 79-Year-Old Female With a Palpable Left Breast Mass

**Figure 9:**
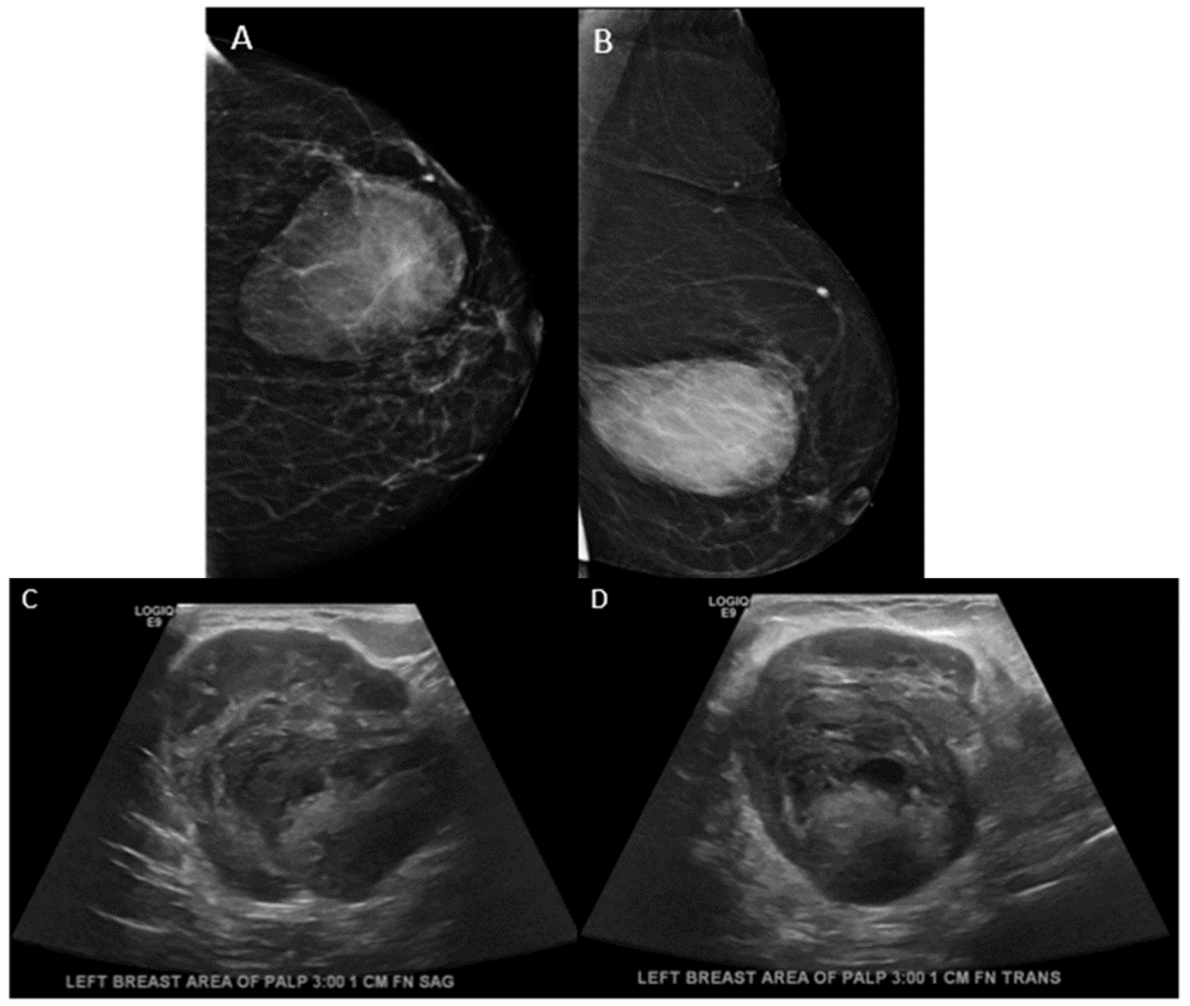
Case 5 initial imaging findings A, B: Craniocaudal and mediolateral oblique tomosynthesis views of the left breast show an oval, high-density mass with circumscribed margins in the central outer left breast corresponding to the area of palpable concern. C, D: Ultrasound grayscale imaging demonstrates a round heterogeneous mass with small central cystic areas and partially circumscribed margins.

A 79-year-old Asian female presented with a palpable mass in her left breast. Mammography revealed a large round to oval mass with circumscribed and indistinct margins in the left central, outer breast measuring up to 5.8 cm (Figure 9D). The ultrasound revealed a large round complex mass with hypoechoic and echogenic areas and microlobulated margins in the area of palpable concern (Figure 9A).

**Figure 10:**
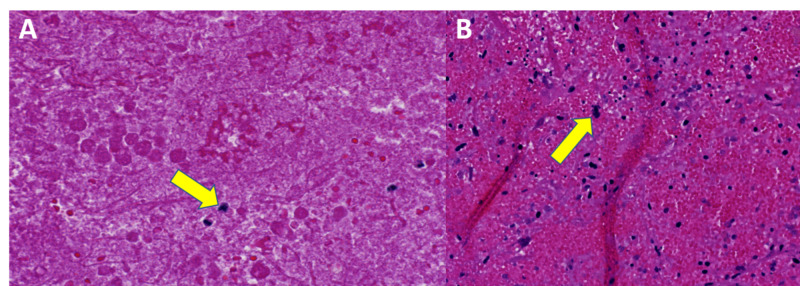
Case 5 initial histopathology attempts A: First biopsy, nonviable, highly pleomorphic cells (yellow arrow) with associated fibrinopurulent exudate. The specimen consists mostly of necrotic highly pleomorphic tumor cell debris. B: Fragments of blood clots and minute scattered collections of atypical cells (yellow arrow). Recommended resampling.

An ultrasound-guided biopsy was performed twice, one month apart, first reporting nonviable highly pleomorphic cells with associated fibrinopurulent exudate (Figure 10A) and then showing fragments of blood clot and minute, scattered collections of atypical cells (Figure 10B).

**Figure 11:**
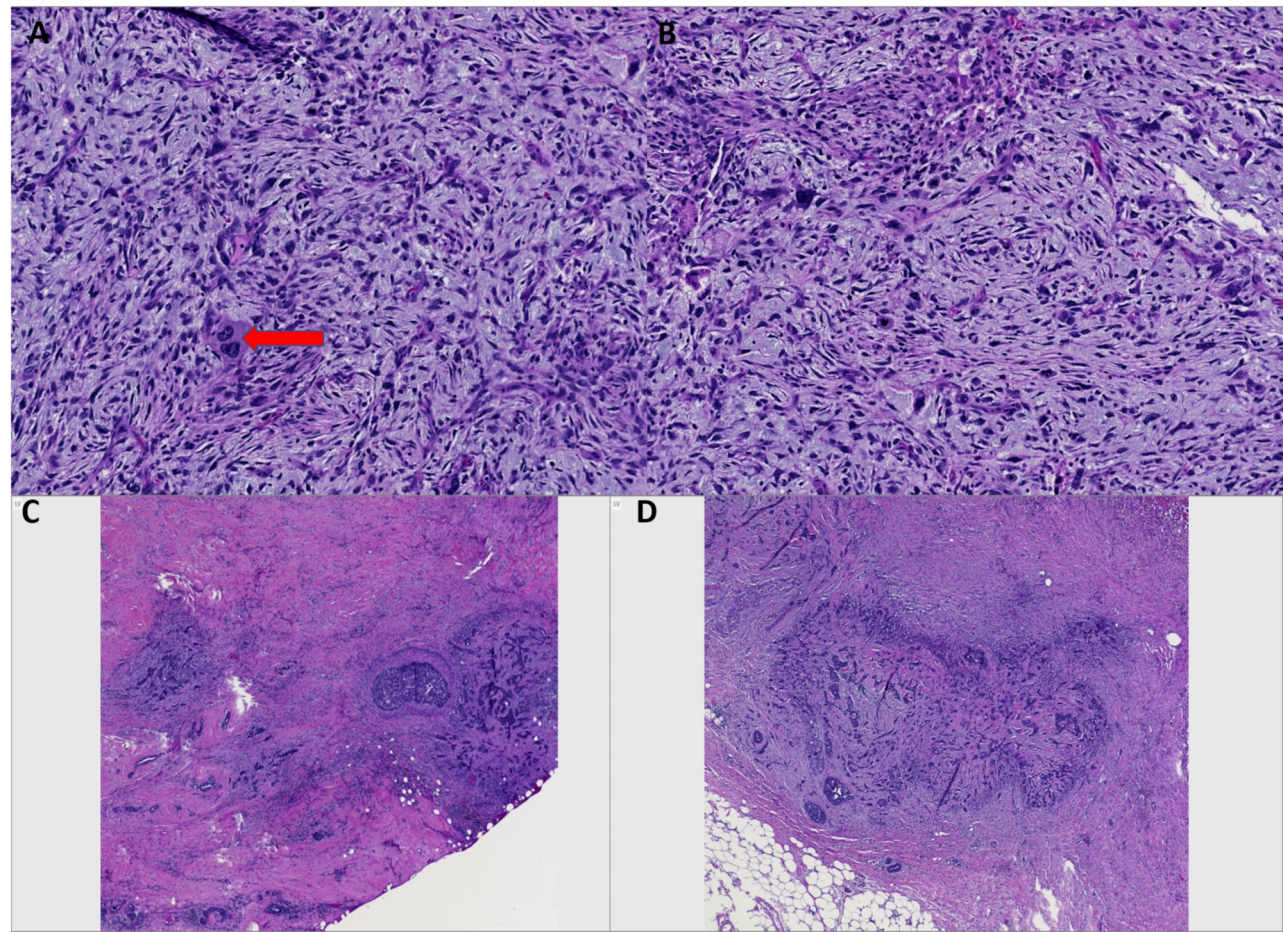
Case 5 excisional biopsy A, B: Images of high-grade spindle cell component composed of highly pleomorphic cells, atypical mitoses with myxoid background. Notice the bizarre binucleated cell in image, A (red arrow), 10x. C, D: Focal areas of more classic high-grade invasive ductal carcinoma and carcinoma in situ.

Due to the presence of a mass radiographically with previous biopsies not revealing viable tissue or tumor cells, again resampling was recommended to obtain viable tissue for an accurate diagnosis. Subsequently, the patient underwent an excisional biopsy that yielded useful material for the diagnosis of metaplastic carcinoma, spindle cell type, with extensive necrosis and hemorrhage (Figures 11A-11D). 

**Figure 12:**
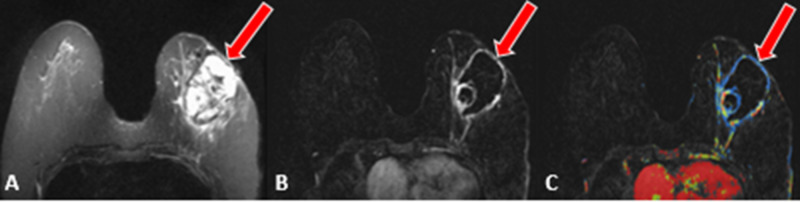
Case 5 breast MRI findings A: Axial T2-weighted image shows a heterogeneous mass with areas of T2 hyperintensity. B, C: Axial T1-weighted fat-suppressed subtracted without (B) and with (C) color show a heterogeneously enhancing centrally necrotic mass in the left breast (red arrows).

A new diagnosis of left breast metaplastic cancer stage III (T3N0M0) triple-negative was subsequently made. MRI of the left breast revealed a large enhancing mass in the outer to central left breast measuring 6.7 x 6.2 x 5cm with necrotic and solid components (Figures 12A-12C).

The PET scan did not show any evidence of metastasis. The patient underwent a modified radical mastectomy with a sentinel lymph node biopsy. Pathologic findings confirmed the diagnosis of metaplastic carcinoma, spindle cell variant, along with areas of poorly differentiated invasive ductal carcinoma-not otherwise specified (NOS), as well as high-grade ductal carcinoma in situ. All lymph nodes submitted were negative for metastatic disease.

## Discussion

Metaplastic carcinoma of the breast is a heterogeneous and diverse group of rare, aggressive malignancies that are characterized by the coexistence of epithelial and non-epithelial components [8]. Several distinctions exist when comparing metaplastic to other histologic subtypes of invasive carcinoma. Metaplastic carcinomas have a more advanced stage at diagnosis. A review of the National Cancer Database from 2006 revealed that patients with MBC had higher rates of stage III and stage IV disease than patients with non-metaplastic carcinomas [9]. Metaplastic carcinomas have a proclivity for hematogenous metastasis but are less likely to have lymphatic involvement [10]. They are more likely to recur and have a reduced time period until recurrence when compared to non-MBC [11].

MBC has a broad histological profile, consisting of a heterogeneous group of malignant neoplasms, with both glandular and non-glandular components, along with mixed epithelial and mesenchymal differentiation. MBC is classified into five subtypes: spindle cell, squamous cell, carcinosarcoma, matrix-producing, and metaplastic carcinoma with osteoclastic giant cells [12]. The most common and most frequently misdiagnosed subtype is spindle cell. The spindle cell subtype is the most difficult to diagnose because of its close resemblance to low-grade sarcomas, phyllodes tumors, or a reactive process such as granulation tissue [13]. For example, Case 4 was initially misdiagnosed as a benign fibroepithelial neoplasm discovered on the ultrasound-guided core biopsy with the correct diagnosis of MBC (spindle call variant) made after surgical excision of the mass. The squamous cell carcinoma subtype displays infiltrating squamous carcinoma with polygonal cells, eosinophilic cytoplasm, and possible keratin pearl formation [14]. The carcinosarcoma subtype is composed of malignant epithelium and malignant stroma. The matrix-producing subtype consists of an apparent carcinoma with a transition to cartilaginous and/or osseous stromal matric lacking a spindle component [15]. Metaplastic carcinoma with the osteoclastic giant cells subtype displays intraductal or infiltration carcinoma contiguous or mixed with spindle cell or sarcomatous stroma plus osteoclastic cells [16]. Although some studies have mentioned the fibromatosis-like subtype of MBC has a slightly better outcome than the other, no definitive research has established any significant difference in overall prognosis solely based on histologic characteristics.

Although metaplastic carcinomas are most commonly negative for estrogen, progesterone, and HER2-like other invasive carcinomas, unique genetic variations and intratumoral heterogeneity cause this tumor type to be more chemoresistant than other breast cancers. Neoadjuvant chemotherapy may be insufficient to halt tumor progression. Multiple studies have demonstrated results coinciding with a limited effect of neoadjuvant treatment, including a 2011 study showing a progression rate of 82% [16]. This highlights the importance of the prompt and accurate diagnosis of MBC. Although immunotherapy is in its infancy, metastatic MBC has been seen to respond completely to checkpoint blockade medications, such as PD-L1 inhibitors, even when initially chemo-refractory [17]. This may be due to the propensity of MBC to express the PD-1 ligand, perhaps as a mechanism to avoid immunodetection and tumor elimination in the immunocompetent host [18].

Many patients with metaplastic breast carcinoma are initially misdiagnosed because it is difficult to classify rare tumors based on heterogeneous imaging characteristics. Similarly, when metaplastic tumors undergo core biopsies, they are histologically difficult to assess because they contain distinct components within the same tumor. In the cases reviewed above, some patients underwent multiple biopsies before the correct diagnosis was reached, and only then did they receive appropriate treatment. The delay in diagnosis may have provided the tumors time to grow and metastasize. Intratumoral heterogeneity leads to sampling bias and highlights the need to obtain an excisional biopsy in the setting of discordance between histopathology and imaging findings. An optimal therapeutic approach remains controversial, however, it appears that a multidisciplinary, targeted approach may be the most effective when it can be promptly initiated.

Loco-regional management with surgery followed by chemoradiation is the current standard of care [19]. Advancements in histopathology, radiomics, and artificial intelligence may speed up the development of new diagnostic and therapeutic strategies to target individual tumors’ unique pathogeneses and ameliorate the need for invasive treatment. Future systemic and intratumoral immunotherapy treatment options may be guided by the identification of the underlying genetic aberration and tumor mutational burden [20].

## Conclusions

The complex nature of this variant of breast cancer leads to difficulty in diagnosis due to sampling bias. The traditional paradigm used to efficiently diagnose high-incidence breast cancers may not account for this characteristic of MBC. These biologically aggressive tumors may express heterogeneous imaging and histologic phenotypes, potentially hampering a timely diagnosis. Red flags include a large, new, or rapidly growing mass that may have some benign imaging features on mammography. Complex, mixed, solid, cystic masses with heterogeneous echogenicity and noncircumscribed margins are some suspicious sonographic features. In these cases where the imaging phenotype is inconsistent with the timeline, it may be useful to obtain breast MRI to look for a high T2 signal consistent with tumor necrosis. Intratumoral heterogeneity may result in a false-negative biopsy and the propensity to develop into an aggressive metastatic disease is high for MBC. If there is any suspicion for MBC based on imaging characteristics, and the initial biopsy is negative for malignancy, it may be advisable to perform an excisional biopsy of the lesion in order to circumvent the significant sampling bias that exists from a small core needle biopsy that may or may not obtain adequate tissue for proper diagnosis. These cases exemplify the importance of and the difficulties in achieving an accurate and timely diagnosis of MBC. Treatment considerations may be tailored to give the patient the best chance of survival, especially if the correct diagnosis of MBC is reached before hematogenous seeding can occur.
